# Online Interactive Platform for COVID-19 Literature Visual Analytics: Platform Development Study

**DOI:** 10.2196/26995

**Published:** 2021-07-16

**Authors:** Addy Moran, Shawn Hampton, Scott Dowson, John Dagdelen, Amalie Trewartha, Gerbrand Ceder, Kristin Persson, Elise Saxon, Andrew Barker, Lauren Charles, Bobbie-Jo Webb-Robertson

**Affiliations:** 1 Pacific Northwest National Laboratory Richland, WA United States; 2 Lawrence Berkeley National Laboratory Berkeley, CA United States; 3 Department of Materials Science and Engineering University of California, Berkeley Berkeley, CA United States

**Keywords:** COVID-19, visual analytics, natural language processing, scientific literature, software, online platform, literature, interactive, publish, research, tool, pattern, usability

## Abstract

**Background:**

Papers on COVID-19 are being published at a high rate and concern many different topics. Innovative tools are needed to aid researchers to find patterns in this vast amount of literature to identify subsets of interest in an automated fashion.

**Objective:**

We present a new online software resource with a friendly user interface that allows users to query and interact with visual representations of relationships between publications.

**Methods:**

We publicly released an application called PLATIPUS (Publication Literature Analysis and Text Interaction Platform for User Studies) that allows researchers to interact with literature supplied by COVIDScholar via a visual analytics platform. This tool contains standard filtering capabilities based on authors, journals, high-level categories, and various research-specific details via natural language processing and dozens of customizable visualizations that dynamically update from a researcher’s query.

**Results:**

PLATIPUS is available online and currently links to over 100,000 publications and is still growing. This application has the potential to transform how COVID-19 researchers use public literature to enable their research.

**Conclusions:**

The PLATIPUS application provides the end user with a variety of ways to search, filter, and visualize over 100,00 COVID-19 publications.

## Introduction

COVID-19 has generated a multitude of challenges for scientific and medical researchers, but one of the unexpected challenges was the pace at which scientific literature emerged. In addition to the continually growing body of research that includes many thousands of publications in a single week, there is also related research on other coronaviruses or comorbidities of interest [1,2]. Computational researchers have been working diligently to assemble this information into minable collections such as CORD-19 [3], CovidScholar [4,5], and LitCovid [6,7]. These data sets are of high value but have limited interaction capabilities. Currently, the primary approach for the scientific community to work with these extremely large corpuses of literature has been through data science–based solutions via search engines and tools that categorize data into facets, which works well for very targeted queries [8,9].

With the onslaught of publications being released to help combat COVID-19, there are multiple solutions to search for information within COVID-19 publications. Examples include the Centers for Disease Control and Prevention’s (CDC) COVID-19 PubMed Search Alert [10], where the user can specify certain criteria and, when a new publication gets released that matches the user’s conditions, the user gets notified. PubMed Search Alert does not provide any support for viewing or searching currently available publications. The CDC also has the PubMed Clinical Queries [11] that allows search by keywords and filter by category, but there are no visualization capabilities, and it returns a simple list of publications. Data-driven visualizations derived from the contents and metadata of these publications can help guide researchers by distilling down the number of publications into a manageable amount while preserving the theme of the query. A newly released tool CoronaCentral [12] offers an improved interface with some visualizations to make searches simpler through a detailed categorization scheme and offers some basic graphics of data summaries based on these categories. The CovidScholar database also helps users with parsing the data via specific tagging classifications and offers a visualization of word embeddings of subsets of papers [4,5]. However, advanced visual analytics of this expanding corpus requires new data science and software solutions. We present a novel platform PLATIPUS (Publication Literature Analysis and Text Interaction Platform for User Studies), which builds on the comprehensive CovidScholar data set and uses visual analytics to give basic and medical researchers a more user-friendly approach to explore their queries of interest. PLATIPUS is publicly available at [13].

## Methods

### Data

The literature presented in PLATIPUS is collected from original publishers in collaboration with the COVIDScholar project at the University of California, Berkeley/Lawrence Berkeley National Laboratory [5]. Articles in COVIDScholar are sourced by a system of dedicated web scrapers, document parsers, databases, and machine learning models that process papers and metadata into a standardized format that is amenable for text mining. The data in COVIDScholar includes a culmination of 19 sources, presented in Textbox 1, and consists of academic preprints, peer-reviewed research papers, book chapters, patents, clinical trial descriptions, and data sets, all of which have been made openly available by the original publishers to advance COVID-19 research. COVIDScholar updates their data multiple times per day and PLATIPUS queries the COVIDScholar database and reingests new articles once a day.

**Main sources of data in COVIDScholar collection.**

**Preprints and non–peer-reviewed articles**

- medRxiv
- bioRxiv
- Preprints.org
- PsyArXiv
- Social Science Research Network
- SocArXiv
- ChemRxiv
- National Bureau of Economic Research

**Peer-reviewed journal articles**

- Elsevier
- PubMed
- CORD-19
- Dimensions

**Book chapters**

- CORD-19

**Patents**

- The Lens

**Clinical trials**

- Dimensions

**Data sets**

- Dimensions

### Text Analytics

PLATIPUS uses a tool called Automated Analytics and Integration of Data (AAID) to assist in the data ingestion and advanced analytic processing of the COVIDScholar data set. AAID uses multiple algorithms to identify key sources of information while taking into account how the meaning of words change based on the context [14]. AAID uses natural language processing methodologies, specifically entity recognition, machine learning, and human-in-the-loop, to augment the data with additional queryable tags [15]. In PLATIPUS, this means augmenting the COVIDScholar data set with tags such as locations, organizations, diseases, diagnostics and analysis, countermeasures, species, and additional context. AAID uses the NiFi data ingestion and processing pipeline that contains a variety of natural language processing methods such as time-weighted penalized logistic regression models, recursive regex, binary bag of words models, and recurrent neural network models, which is described in detail in Multimedia Appendix 1 Figure S1. The vectorization of the text was based on a bag of words approach. For the clustering visualizations, a k-means default method was used. The analytic capabilities of the AAID pipeline continue to grow to use transformer deep learning classifiers and implement methods to identify anomalies and abnormal characteristics [16].

As of May 2021, there are 159,797 articles that are parsed into various filters. At the top level are authors (n=564,845), categories (n=7), context (n=41), countermeasures (n=28), diagnostics and assay (n=19), disease (n=265), journal (n=11,412), locations (n=365), tags (n=7), species (n=76), and chemicals (n=175). Authors are associated with the publications, and therefore, there are hundreds. For selection purposes, the authors are sorted in order from the most to least prevalent. There are presently seven core categories (treatment, prevention, mechanism, diagnosis, epidemic forecasting, transmission, and case report). Under context, there are 41 groupings associated with the primary context of the article (eg, disease severity or transmission event). Countermeasures are approaches taken against the disease (eg, treatment, vaccine, or awareness campaign). The diagnostics and assay groupings contain the platforms associated with the article, such as transcriptomics or x-rays. Disease is again a broad category where the most prevalent is a categorization of human or animal disease but other specific associated syndrome or special notes are captured here. Journal, similar to author, is a large group of the virtual location of the publication online. Location is a physical location at which the research or case study is conducted for publication, which are extracted using resources from the National Geospatial-Intelligence Agency and United States Geological Survey [17,18]. There are 76 species, the most prevalent being human, rodents, and swine, and 175 chemicals captured that are associated with the manuscripts.

### Application Development

**Figure 1:**
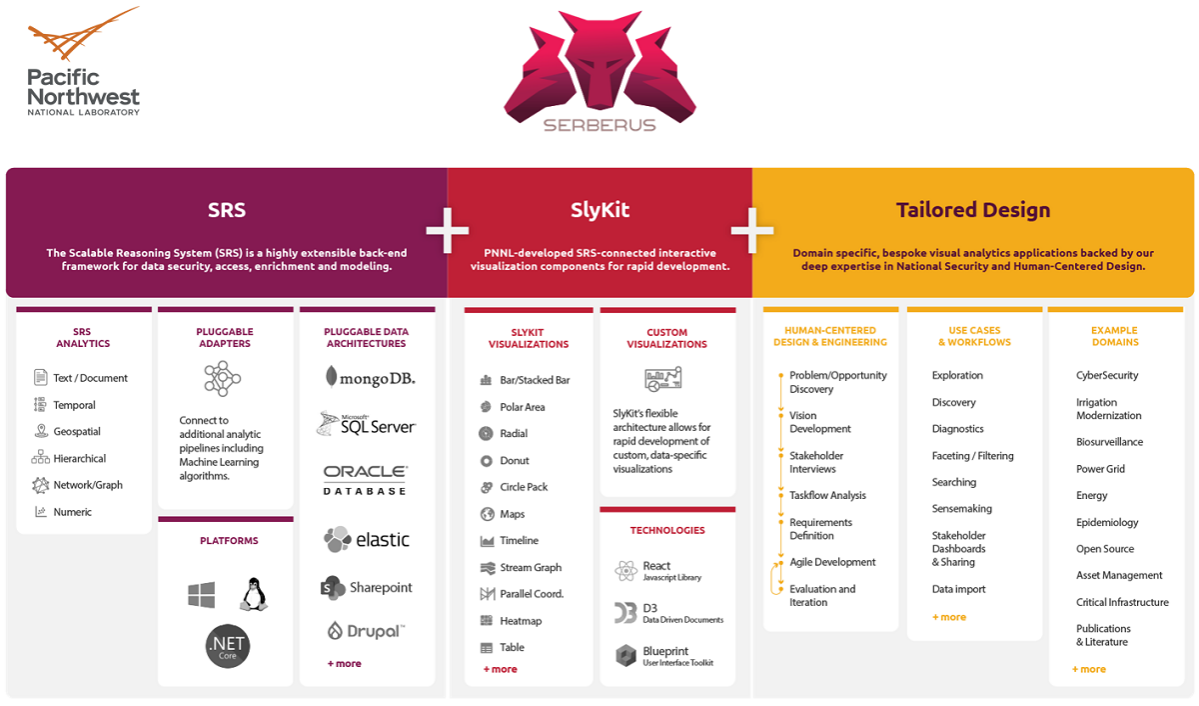
Description of the SERBERUS application full capability components. PNNL: Pacific Northwest National Laboratory.

PLATIPUS is built on top of the SERBERUS application, which is an end-to-end software solution that rapidly builds visual analytic web applications (Figure 1). Powered by the Scalable Reasoning System (SRS) [19] on the back end and a flexible user interface toolkit on the front end, and drawing from expertise from a user experience and design team, this system is designed for custom solutions that can be readily constructed to support data exploration, discovery, and understanding.

The PLATIPUS application provides the end user with a variety of ways to search and filter over 100,000 COVID-19 publications. Since PLATIPUS is built on top of SRS and Slykit, PLATIPUS will continue to evolve and grow with new visualizations and features as SRS and Slykit advances. As of May 2021, PLATIPUS allows the user to filter on locations, categories, authors, organization, disease, diagnostics and analysis, countermeasures, species, and additional context as well as a timeline. The visualizations that are currently available are circle pack, cluster pack, donut graphs, edge-based graph, line chart, matrix, metrics, paracord, table, text clusters, treemap, and timeline described in Textbox 2. The first 10 of these visualizations are at the center of the dashboard and can be assembled based on user choice (one, two, three, etc) all in the view. The timeline visualization is maintained across the top of the user interface. At any time during the filtering and searching process, the user can access a high-level overview of an individual publication, which includes the abstract, information about the authors, tags and categories, and the journal where it was published as well as a direct link to the full publication. Once the user filters down to a subset of publications of interest, they can export the list of publications as a CSV file.

**PLATIPUS (Publication Literature Analysis and Text Interaction Platform for User Studies) core visualizations.**

**Circle pack**

Relative-sized circles of various metadata fields that supports up to three levels (ie, categories→disease→locations)

**Cluster graph**

Primary properties are clustered into nodes, which are resized based on connection count.

**Donut graphs**

Data separated based on various properties in a donut circle view where sizes within the donut are relative to frequency

**Edge-based graph**

Primary property is connected via nodes from a defined link property, which can be filtered based on the number of connections.

**Line chart**

Multiline chart customized to property selected, data binning, color, and aggregation

**Matrix**

A 2D grid that shows the aggregations between two properties

**Metrics**

High-level summary of the data selected

**Paracord**

Links properties to find connection between metadata, especially useful to find single unique connections

**Table**

Read-only table format to sort and limit the items being viewed

**Text clusters**

Groups keywords to place documents into common clusters

**Timeline**

Bar graph to display metadata over time

**Treemap**

Recursive drill down into subgroups from a primary group

## Results

**Figure 2:**
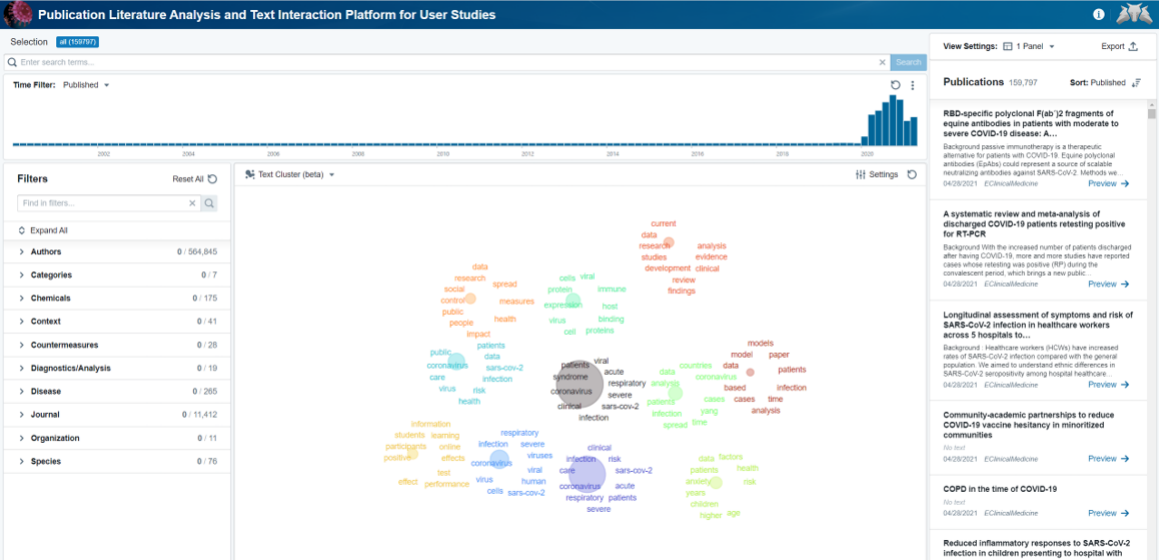
Screenshot of the primary PLATIPUS (Publication Literature Analysis and Text Interaction Platform for User Studies) application page from which the user begins queries and visual interactive activities.

The application allows the user to search by keyword, filter by various tags, select a time range, and visualize the tags and other document properties on innovative graphs and visualizations. Figure 2 shows the home screen of PLATIPUS, which is showing the test clustering view of the full set of COVID-19–related publication literature. PLATIPUS is broken into multiple panels: the search bar on top center, the timeline for filtering articles by date in the center, the filters associated with the annotated data (eg, authors or journals) on the left, the visualization panel (9 total options) in the bottom center, and the article panel (right).

**Figure 3:**
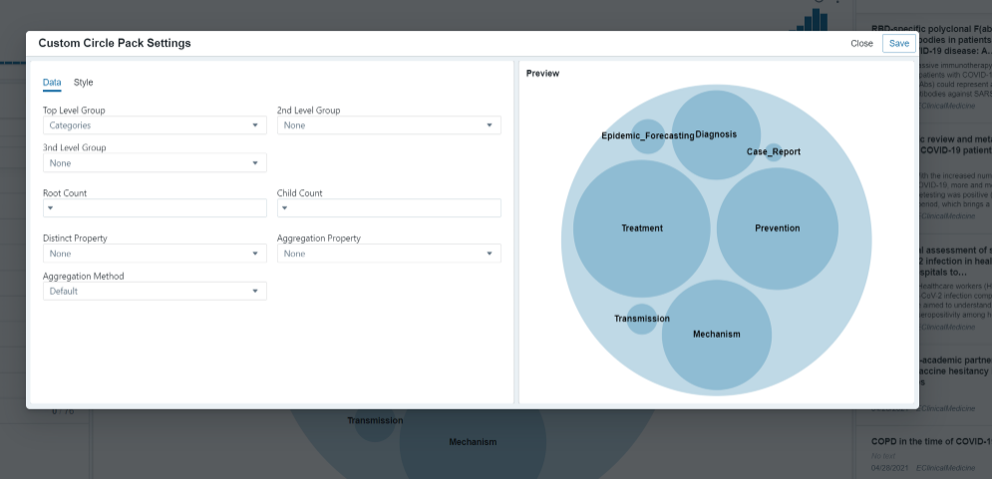
Example of the visualization customization component.

One of the key features of PLATIPUS is the numerous approaches that can be taken to visualize the data. Figure 3 highlights one alternative to the text cluster in Figure 2 (custom circle pack) and how each visualization can be modified to show the specific information of interest to the user. The custom circle pack is driven from the filters on the left-hand side and allows quick views of the overall distribution of this information. For example, for all the COVID-19–related articles in PLATIPUS, we see the majority fall into four core categories: diagnosis, treatment, prevention, and mechanism.

**Figure 4:**
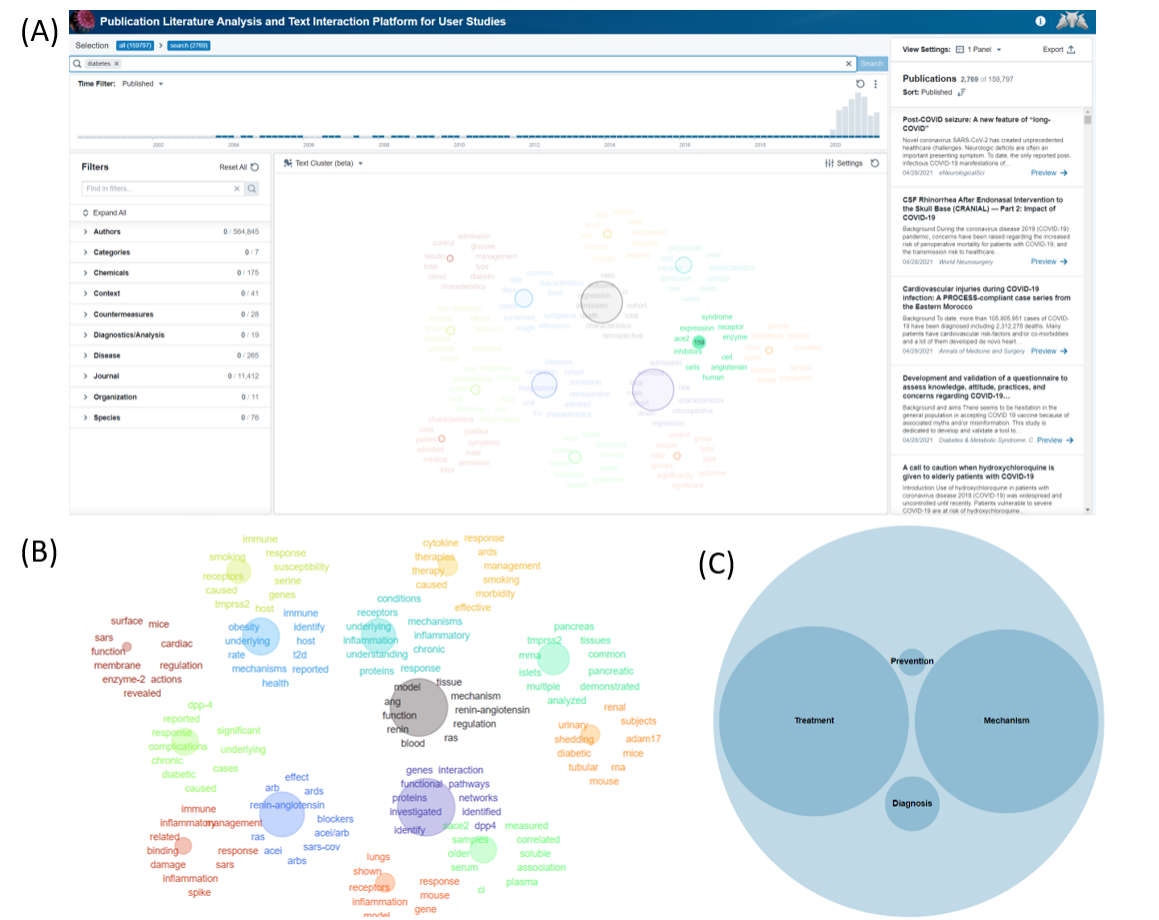
(A) View and selection based on a search for the term “diabetes” where a cluster associated with angiotensin-converting enzyme 2 (ACE2) is identified, with (B) and (C) as alternate visualizations of the results after selection of the cluster including ACE2.

**Figure 5:**
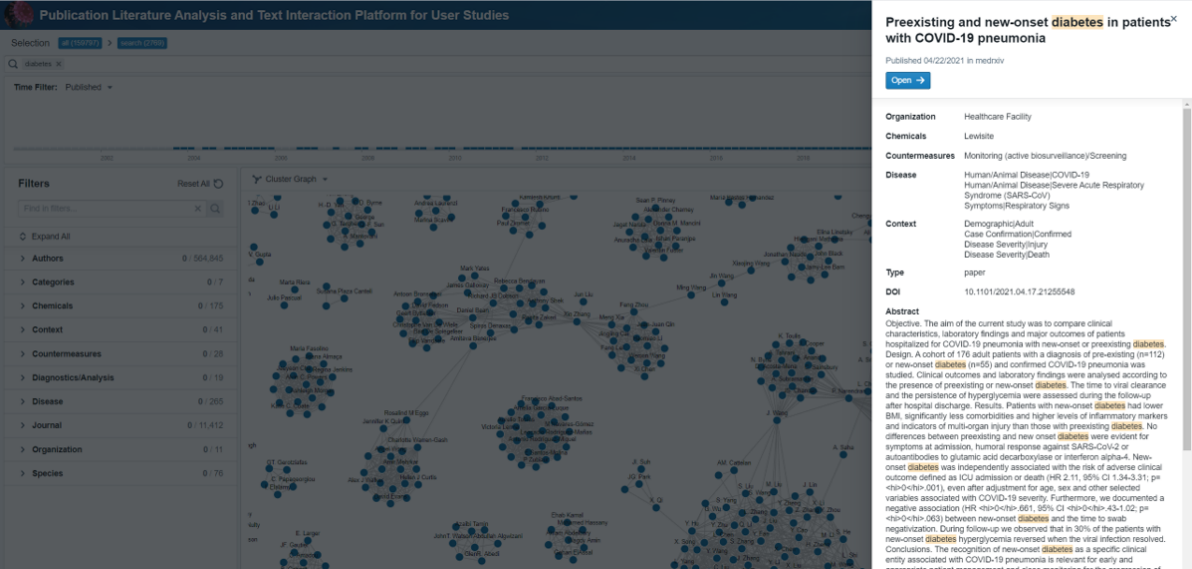
High-level view of a paper, which includes information about COVID-19 and diabetes.

To further explore the functionality of the PLATIPUS application, we demonstrate an example via a case study. There has been significant evaluation of comorbidities such as diabetes on the prognostic response of patients with COVID-19 [20-23]. In this case study, the search of the term “diabetes” in PLATIPUS returns 2769 articles from the originating 159,797, as of May 2021 (Figure 4A). However, this number is too many for a researcher to search through manually. Often the researcher performing the search will select the first few to read in more detail by perusing abstracts or other down-select criteria. This method is still an option within PLATIPUS, as the articles and abstracts are displayed on the right-hand side of the application. A benefit of PLATIPUS is the additional clustering visualization of articles that goes beyond the standard sorting function available in most publication search engines. By evaluating the clusters located in the center of the application (Figure 4A), a researcher interested in the putative receptor angiotensin-converting enzyme 2 can see this is a key cluster in the visualization. Selecting this cluster reduces the literature from 2769 to 159 articles. PLATIPUS then allows the researcher to observe clusters of articles within this new refined query (Figure 4B). The researcher can either narrow down further this way or, as an alternative, can filter articles within the defined facets using a variety of methods (custom circle pack shown in Figure 4C). Within this refined search, the user can view any of the publications via the reading pane. By choosing preview, a publication will open to allow researchers to view the full abstract and associated metadata, and link to the full text, if available, as seen in Figure 5 [24]. Alternatively, on the left side of Figure 4A, there are predefined filters, which include subsets such as “Diagnostics” or “Disease” as an alternate approach to filtering the data. The researcher can also export the metadata from selected documents as a CSV for review in the future.

**Figure 6:**
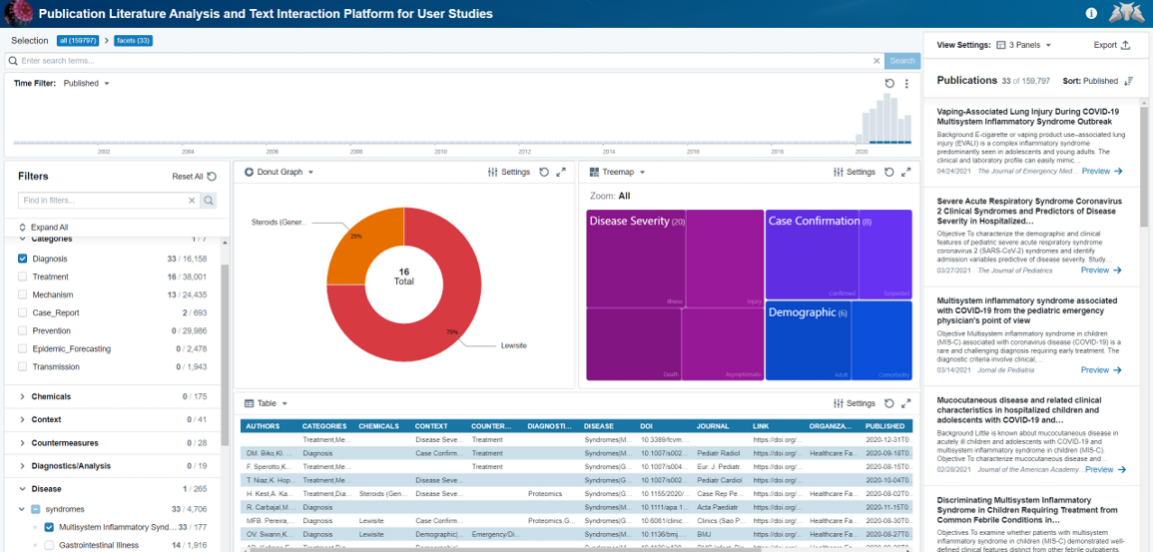
View and selection based on tagging capability drilling into tags of multisystem inflammatory syndrome and diagnosis.

The diabetes example is a visual analytics exploration of a relatively open question, but PLATIPUS also supports direct medical queries using the valuable tagging that is supplied via the AAID pipeline associated with the CovidScholar data. For example, as seen in Figure 6, we applied two filters to find literature that can help with the diagnosis of “Multisystem Inflammatory Syndrome” and “Diagnosis.” Multisystem inflammatory syndrome is a new clinical condition due to a cytokine storm associated with COVID-19 that causes inflammation and organ failure [25]. In PLATIPUS, the first filter selected is “Multisystem Inflammatory Syndrome,” which reduces the data set to 177 manuscripts. This is further refined into a small set based on the selection of “Diagnosis,” which reduces to 33 articles, visible on the left-hand side of Figure 6. The visualizations in this case are tailored to give context of the type of chemical information that is identified from the paper, which may give further insight into how to down-select. The treemap allows the researcher to see the 33 articles that are categorized based on the information of this specific query. Evaluating the 33 articles quickly points to an environmental component of multisystem inflammatory syndrome [26-29].

## Discussion

### Principal Results

The primary manner the scientific community interacts with scientific literature has, up until recently, not changed in decades. COVID-19 has brought to the forefront of research the challenge of mining literature versus identification of potential articles of interest to a user by keyword searches. To date, PLATIPUS has performed text analytics and clusters, and has visualized nearly 160,000 articles related to COVID-19, and it automatically updates as new documents are added to COVIDScholar. The application uses state-of-the-art natural language processing (AAID) to provide insight and unique ways to filter and understand the data. PLATIPUS aims to decrease time spent looking through pages of articles by providing the user with multiple ways to search, filter, and view the data. The PLATIPUS application focuses on taking the large amount of literature related to COVID-19 and displaying keywords, categories, and other metadata to allow a user to quickly find relevant information captured by COVIDScholar.

### Limitations

PLATIPUS was designed to assist in searching a multitude of COVID-19 publications efficiently, so the user can either find their answer using the visualizations, searching, and drill down capabilities or find a document that will assist in their search. Therefore, PLATIPUS does not support saving views or searches, as it was designed to be a visual analytics search engine and visual table of contents. Additional limitations include the suggestion of the *optimal* visualization based on a query. PLATIPUS allows the users to toggle through visualizations and select those that are of the most utility. Additions to PLATIPUS in the future may be a more guided visualization experience based on the size and complexity of the literature returned from a query. As of March 2021, PLATIPUS does not support finding similar articles to a single selection, but we expect this feature will be available in the future.

## Appendix

Multimedia Appendix 1 PLATIPUS NiFi pipeline diagram.

## Abbreviations

AAID: Automated Analytics and Integration of Data
CARES: Coronavirus Aid, Relief, and Economic Security
CDC: Centers for Disease Control and Prevention
DOE: Department of Energy
PLATIPUS: Publication Literature Analysis and Text Interaction Platform for User Studies
SRS: Scalable Reasoning System
