# Supplementary figures and images for: Online Interactive Platform for COVID-19 Literature Visual Analytics: Platform Development Study
Source: J Med Internet Res. 2021 Jul 16;23(7):e26995. doi: 10.2196/26995 (PMC8288648; doi:10.2196/26995)

Platipus NiFi Pipeline Diagram

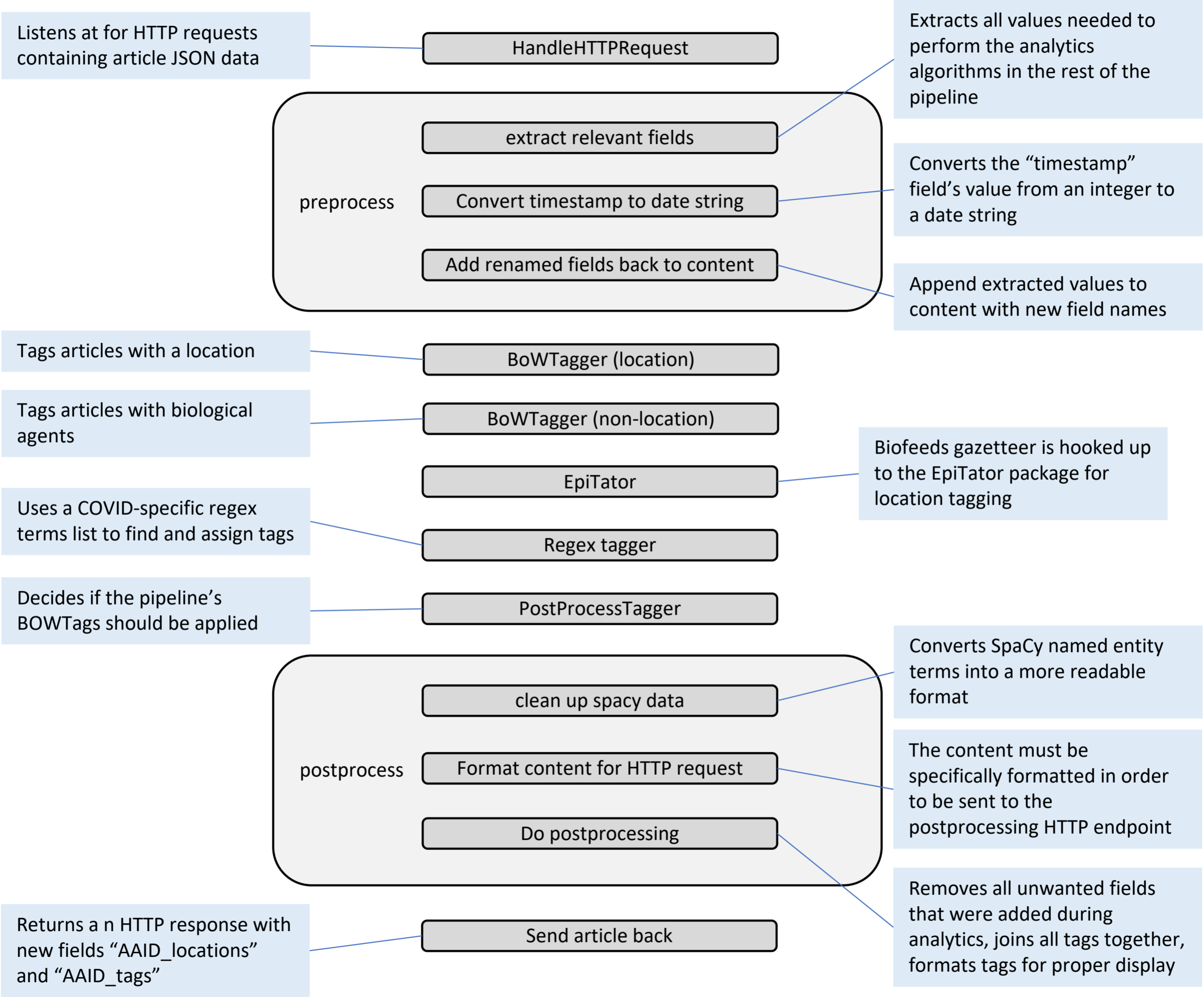

Supplement: Multimedia Appendix 1 [file jmir_v23i7e26995_app1.pdf]
